# Circulating Tumor DNA in Neurofibromatosis Type 1: Translating Molecular Discovery into Clinical Surveillance

**DOI:** 10.3390/diagnostics16132063

**Published:** 2026-07-01

**Authors:** Joanne Vanessa Vargas, Valeria Tosello, Giulia Pigato, Stefano Indraccolo, Federica Chiara

**Affiliations:** 1 Department of Surgery, Oncology and Gastroenterology, University of Padova, 35121 Padua, Italy; joannevanessa.vargas@unipd.it (J.V.V.); stefano.indraccolo@unipd.it (S.I.); 2 Basic and Translational Oncology Unit, Veneto Institute of Oncology IOV-IRCCS, 35121 Padua, Italy; valeria.tosello@iov.veneto.it (V.T.); giulia.pigato@iov.veneto.it (G.P.)

**Keywords:** neurofibromatosis type 1 (NF1), liquid biopsy, circulating tumor DNA (ctDNA), malignant peripheral nerve sheath tumor (MPNST), early detection, fragmentomics, methylomics, precision medicine

## Abstract

Neurofibromatosis type 1 (NF1) is a genetic tumor predisposition syndrome characterized by a substantial risk of developing peripheral nerve sheath tumors, including malignant peripheral nerve sheath tumors (MPNSTs), which occur in 8–13% of patients. Approximately 50% arise from plexiform neurofibromas (PNs) and 40% develop de novo, making them a major cause of premature mortality. Current clinical management is limited by the intrinsic shortcomings of standard imaging modalities: magnetic resonance imaging (MRI) and positron emission tomography/computed tomography (PET/CT), and tissue biopsy in distinguishing benign PNs from early malignant transformation, which remains a major clinical challenge. This progression follows a stepwise molecular continuum marked by cumulative genetic alterations and widespread epigenetic dysregulation. In this setting, liquid biopsy has emerged as a promising non-invasive approach to help fill these diagnostic gaps by enabling real-time molecular monitoring through the analysis of circulating tumor DNA (ctDNA) and other blood-based biomarkers. This review examines the current evidence supporting liquid biopsy applications in NF1 management, including early detection of MPNST, discrimination between benign and malignant lesions, mutational profiling for therapeutic targeting, and disease monitoring before and during treatment. We also discuss the current evidence on fragmentomics, methylomics and driver mutation profiling as tools to distinguish PNs from MPNSTs. Recent evidence suggests that liquid biopsy may help detect molecular changes associated with malignant transformation before clear clinical signs emerge, potentially opening an important window for intervention and supporting a shift towards a more molecularly informed surveillance model. Finally, this review considers the possible extension of liquid biopsy to other tumor types, including *NF1*-deficient breast cancer, and outlines a future management framework aimed at improving early diagnosis and personalized therapeutic intervention in this high-risk population.

## 1. Introduction

Neurofibromatosis type 1 (NF1) is a common autosomal dominant tumor predisposition syndrome, affecting approximately 1 in 2500–3000 individuals worldwide [1,2]. It is classified as a RASopathy, a group of genetic conditions caused by germline mutations in the RAS-MAPK signaling pathway [3,4]. The disorder results from loss-of-function mutations in the *NF1* tumor suppressor gene located on chromosome 17q11.2, which encodes the protein neurofibromin [5]. Neurofibromin acts as a critical negative regulator by accelerating the conversion of active RAS-GTP to its inactive RAS-GDP state [6,7,8]. As a result, its loss leads to constitutive RAS hyperactivation, driving uncontrolled cellular proliferation and survival [9]. NF1 shows remarkable phenotypic heterogeneity, even among family members carrying the same mutation [10,11]. Although the hallmark of the disease is the development of multiple neurofibromas—benign nerve sheath tumors arising from Schwann cell lineage progenitors—NF1 is, in reality, a multisystemic syndrome. Beyond its oncologic manifestations, patients experience a wide range of complications, as *NF1* is defined by a constellation of diagnostic features reflecting the involvement of neural crest-derived tissues. Café-au-lait macules are usually the earliest manifestation, often appearing in early childhood, and are characterized by sharply demarcated, uniformly pigmented light-brown patches [12]. Axillary and inguinal freckling typically develop later and consist of smaller hyperpigmented macules localized to intertriginous areas [13,14].

Ophthalmologic findings include Lisch nodules—benign melanocytic hamartomas of the iris—which are highly specific for NF1 and increase in frequency with age [15]. Skeletal abnormalities such as tibial dysplasia, pseudoarthrosis, scoliosis, and sphenoid wing dysplasia reflect mesenchymal and developmental involvement [16]. Vasculopathies, including renal artery stenosis and moyamoya syndrome, further illustrate the systemic nature of the disorder [17,18].

Neurocognitive impairment, attention-deficit disorders, and learning disabilities affect a substantial proportion of patients and contribute significantly to long-term morbidity, underscoring that NF1 is not solely a tumor predisposition syndrome but a complex neurodevelopmental condition [19,20].

This complexity necessitates a multidisciplinary approach to management, as these manifestations markedly reduce quality of life and increase lifelong morbidity [21].

It is also important to recognize that NF1 represents not only a rare genetic disorder but also a human model of RAS-driven tumorigenesis across multiple tissues. Population-based cohort studies have shown that individuals with NF1 carry a significantly increased lifetime cancer risk compared with the general population, particularly during childhood and early adulthood. In childhood, the increased tumor incidence mainly concerns optic pathway gliomas and other low-grade gliomas, which represent the most characteristic pediatric NF1-associated tumors. Less frequent pediatric malignancies, including rhabdomyosarcoma and juvenile myelomonocytic leukemia, have also been reported, whereas malignant peripheral nerve sheath tumors (MPNSTs) become more clinically relevant from adolescence and young adulthood. This systemic oncologic vulnerability reflects the central role of neurofibromin loss in maintaining RAS pathway homeostasis across diverse cellular contexts [22,23,24,25,26]: NF1 predisposes to other central nervous system tumors, pheochromocytomas and paragangliomas, gastrointestinal stromal tumors (GISTs), and early-onset breast cancer [27,28,29,30,31,32]. Women with NF1 have a markedly increased risk of breast cancer before the age of 50, with up to a fivefold higher risk compared to the general population. There is important evidence that NF1 is associated with relatively poor breast cancer survival [33]. These observations have led to recommendations for earlier and intensified breast surveillance in affected women.

In particular, NF1-associated GISTs typically arise in the small intestine, are frequently multifocal, and characteristically lack activating *KIT* or *PDGFRA* (PDGF Receptor Alpha) mutations, suggesting a pathogenetic mechanism more directly linked to RAS pathway dysregulation than to canonical receptor tyrosine kinase activation [34,35]. This molecular distinction further emphasizes NF1 as a unique biological framework for investigating RAS-dependent oncogenesis across tissues.

The primary concern in the longitudinal care of NF1 patients is the development of Peripheral Nerve Sheath Tumors (PNSTs), which occur along a pathological continuum [36]. Plexiform neurofibromas (PNs), affecting up to 50% of individuals, are often congenital and grow along major nerve plexuses [37,38]. Although histologically benign, PNs can be life-threatening due to their infiltrative growth, causing compression of vital organs, severe pain, and disfigurement [14,37].

The most devastating complication is the malignant transformation of PNs into MPNSTs, which occur in 8–13% of patients with NF1. In the broader MPNST literature, approximately 50% of cases arise from pre-existing neurofibromas, around 40% develop de novo, and approximately 10% are radiation-associated [39]. MPNSTs are highly aggressive soft-tissue sarcomas and represent the leading cause of premature mortality in NF1, with a dismal 5-year survival rate ranging between 20% and 50% [40]. This transition is not always direct; rather, it often proceeds through a pre-malignant intermediate state known as Atypical Neurofibromatous Neoplasm of Uncertain Biologic Potential (ANNUBP) [41], characterized by increased cellularity and cytological atypia but lacking definitive histological features of malignancy. At the molecular level, this progression reflects a multistep evolutionary process driven by the sequential acquisition of additional somatic alterations beyond *NF1* loss. Frequent events include *CDKN2A* deletion, *TP53* mutations, and inactivation of components of the Polycomb Repressive Complex 2 (PRC2), such as *SUZ12* or *EED*. Loss of PRC2 function results in depletion of H3K27 trimethylation, leading to widespread epigenetic reprogramming, genomic instability, and aggressive tumor behavior [36]. These alterations mirror principles of clonal evolution described in other solid tumors, positioning NF1-associated sarcomagenesis as a valuable model for studying malignant transformation [36,42]. Identifying the precise moment of this transition is clinically crucial, as it determines the therapeutic approach: conservative observation or targeted therapies (such as MEK inhibitors) [43] for PNs versus radical, wide-margin resection for MPNSTs [44,45,46].

At present, clinical management is heavily constrained by the limitations of standard-of-care diagnostics. Surveillance protocols rely mainly on magnetic resonance imaging (MRI) and Positron Emission Tomography/Computed Tomography (PET/CT) [47]. Although MRI provides excellent anatomical detail, it often struggles to distinguish benign PNs, pre-malignant ANNUBPs, and early-stage MPNSTs because of overlapping radiological features such as similar T2 signal intensity and enhancement patterns. 18F-Fluorodeoxyglucose (18F-FDG) PET/CT offers improved sensitivity to metabolic changes through standardized uptake value (SUV) measurements; however, a substantial SUV overlap exists between atypical neurofibromas and low-grade MPNSTs, limiting its specificity [48,49,50,51]. In addition, repeated PET/CT scans expose patients—already at increased risk for radiation-induced secondary malignancies—to cumulative radiation doses, raising concerns in long-term surveillance [52]. Critically, neither modality captures the molecular alterations driving malignant transformation, nor do they reflect real-time tumor evolution or treatment response at the genomic level [53,54].

When clinical evaluation or imaging raises suspicion of malignant transformation, histopathological confirmation through tissue biopsy remains mandatory in current clinical practice. Despite advances in imaging modalities, tissue diagnosis continues to represent the gold standard for differentiating benign plexiform neurofibromas, ANNUBP, and MPNST. However, while indispensable for definitive diagnosis, this approach is associated with significant limitations in the context of NF1 [14]. First, PNs display marked spatial heterogeneity, with areas of high-grade transformation often confined to small focal regions within large, predominantly benign lesions. As a consequence, a needle biopsy may miss these critical foci, resulting in false-negative findings due to sampling bias [53]. Second, MPNSTs frequently contain necrotic areas, further complicating histological interpretation and increasing the risk of non-diagnostic samples [45]. Third, tumors closely associated with major nerve plexuses pose substantial procedural risks, including permanent nerve injury and neurological deficit [38,45]. Finally, even when diagnostic tissue is obtained, immunohistochemical markers such as Ki-67 and S100 show considerable overlap between atypical neurofibromas and low-grade MPNSTs, limiting their diagnostic utility [42].

Taken together, these limitations create critical diagnostic gaps that delay intervention, often leading to MPNST diagnosis only after progression to advanced or metastatic stages [55]. This clinical reality highlights the urgent need for a complementary surveillance tool capable of capturing tumor dynamics in real time, without the constraints of localized sampling or radiation exposure. Given the predictable germline background combined with stochastic somatic evolution, NF1 offers a rare opportunity to investigate tumor initiation, progression, and malignant transformation within a defined genetic landscape. In this setting, surveillance strategies able to capture molecular dynamics rather than static anatomical changes become particularly relevant.

In this context, minimally invasive approaches such as liquid biopsy—primarily the analysis of circulating tumor DNA (ctDNA)—have emerged as a transformative solution. By capturing tumor-derived genomic fragments directly from the bloodstream, liquid biopsy offers a non-invasive, molecular snapshot of the overall tumor landscape, thereby overcoming several limitations of localized tissue samples. Beyond simple mutation detection, advancements in cell-free DNA (cfDNA) fragmentomics, including the analysis of fragment length patterns and end motifs, and methylation markers have opened new possibilities for distinguishing non-malignant, pre-malignant, and malignant states with high accuracy [53].

The aim of this review is to examine the current evidence for liquid biopsy in NF1 management. We will discuss the molecular pathogenesis underlying the neurofibroma-to-sarcoma transition, analyze the technical challenges involved in detecting low-fraction tumor signals, and evaluate how the translation of these molecular insights into clinical surveillance protocols may offer a crucial window for earlier, life-saving intervention.

## 2. The Molecular Roadmap from Neurofibroma to MPNST

The malignant transformation of PNs into MPNSTs is now understood as a stepwise evolutionary process driven by sequential genomic alterations and progressive epigenetic reprogramming, rather than as an abrupt binary event [42,56]. Defining this continuum is essential, as it provides the pathological and molecular framework through which transformation-associated lesions can be recognized, staged, and biologically interpreted.

At the core of this process lies the *NF1* gene itself. The disease is initiated by a germline loss-of-function mutation in the *NF1* gene, resulting in systemic haploinsufficiency of neurofibromin [6,7,56]. However, tumorigenesis requires a second hit, namely the somatic inactivation of the remaining wild-type allele within Schwann cell precursors, leading to complete loss of neurofibromin function and hyperactivation of RAS-MAPK signaling [57,58]. Whole-genome sequencing studies have consistently shown that PNs harbor biallelic *NF1* loss, most commonly through loss of heterozygosity or somatic mutation [14,39]. Yet, despite this strong oncogenic signaling input, *NF1* loss alone is not sufficient to confer malignant behavior.

**Figure 1:**
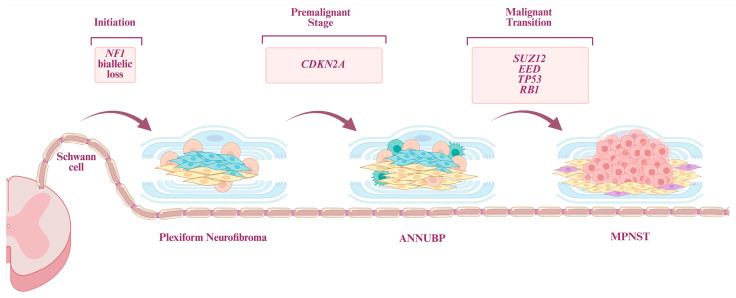
Schematic representation of the stepwise molecular progression of NF1-associated peripheral nerve sheath tumors. Biallelic *NF1* loss in the Schwann cell lineage underlies the development of plexiform neurofibroma (PN). Subsequent *CDKN2A* loss is associated with progression toward atypical neurofibromatous neoplasm of uncertain biologic potential (ANNUBP), whereas additional alterations involving *SUZ12*, *EED*, *TP53* and *RB1* accompany malignant transformation to malignant peripheral nerve sheath tumor (MPNST). This figure summarizes the main molecular events discussed in the NF1 tumor continuum. Created with BioRender.com.

Indeed, the biological consequences of *NF1* inactivation go well beyond cell-autonomous proliferation. Hyperactive RAS signaling profoundly reshapes the tumor microenvironment, promoting the recruitment of mast cells, fibroblasts, and other immune and stromal populations through paracrine mediators such as stem cell factor (SCF) and platelet-derived growth factor (PDGF) [59,60]. This stromal ecosystem contributes to tumor maintenance and therapeutic resistance and is now recognized as a central feature of PN biology [61]. Nevertheless, even within this permissive and chronically activated context, benign PNs remain remarkably genomically stable, with low proliferative indices and a striking paucity of recurrent secondary driver alterations [57,62]. For this reason, the transition from PN to malignancy cannot be explained by *NF1* loss alone but rather reflects the subsequent acquisition of additional molecular events that progressively dismantle normal growth control. A schematic overview of this stepwise progression is shown in Figure 1.

Among these, homozygous deletion of the *CDKN2A/B* locus on chromosome 9p21 represents one of the earliest and most reproducible alterations associated with progression [57,63,64]. The *CDKN2A* locus encodes p53 pathways, respectively [65]. Loss of this locus disrupts cell-cycle arrest and apoptotic control, allowing cells to bypass senescence and tolerate the accumulation of further oncogenic lesions [66,67].

Importantly, *CDKN2A/B* deletion is strongly enriched in ANNUBPs, which are widely regarded as intermediate lesions bridging benign PN and overt MPNST [63,68,69]. Rather than reflecting a diffuse mutational burden, *CDKN2A/B* inactivation in *NF1*-associated precursor lesions is more precisely represented by copy-number loss affecting the 9p21.3 locus, which may appear as a focal *CDKN2A/B* deletion or as part of broader 9p alterations depending on tumor stage, sample composition, and analytical resolution. This makes *CDKN2A/B* loss amenable to detection through array-based copy-number profiling, sequencing approaches able to infer gene dosage, or droplet digital PCR [69,70]. This observation has direct translational relevance, as *CDKN2A* status represents a binary, actionable molecular marker that may help stratify lesions by malignant potential. In the context of liquid biopsy, detection of *CDKN2A* loss in ctDNA could serve as an early sentinel event, signaling the onset of malignant transformation and prompting intensified surveillance or therapeutic intervention [71,72]. Thus, *CDKN2A* emerges not only as a prognostic biomarker, but as a high-priority molecular target for real-time, non-invasive monitoring of disease evolution in NF1 patients.

A further defining step toward malignancy is the inactivation of PRC2, most commonly through loss-of-function mutations in *SUZ12* or *EED* [64,73]. PRC2 catalyzes trimethylation of histone H3 at lysine 27 (H3K27me3), a repressive chromatin mark required to maintain lineage fidelity and suppress inappropriate transcriptional programs [74,75]. Loss of PRC2 function has major biological consequences, including global depletion of H3K27me3, widespread transcriptional derepression, altered chromatin accessibility, and increased genomic instability [76,77,78]. Importantly, these consequences are also reflected at the level of DNA methylation in tumor tissue. Methylation-based profiling has shown that peripheral nerve sheath tumors segregate into biologically meaningful epigenetic classes, further refining the continuum from benign neurofibroma to overt MPNST. In Röhrich et al., benign lesions and low-grade tumors clustered within MeGroup 1, whereas atypical neurofibromas and low-grade MPNSTs were largely indistinguishable at the methylation level and shared frequent *CDKN2A* loss, supporting the existence of an intermediate precursor state. Among high-grade MPNSTs, two principal methylation groups emerged: a predominant subgroup associated with loss of H3K27me3 and a smaller subgroup, often including spinal or paraspinal tumors, that retained H3K27me3 [79]. These findings were further refined by Lyskjær et al., who showed that MeGroup 4 is enriched for histologically classical MPNSTs with loss of H3K27me3, whereas MeGroup 5 comprises tumors with non-classical morphology that more often retain H3K27me3 and cluster more closely with undifferentiated pleomorphic sarcomas. Consistent with this heterogeneity, loss of H3K27me3 was observed in 38% of MPNSTs overall, but in 76% of histologically classical cases, compared with 23% of tumors with heterologous elements and 14% of morphologically ambiguous cases [80].

Overall, these data indicate that PRC2 disruption is not simply inferred from loss of a histone mark, but is embedded within broader tissue-level epigenetic reprogramming that accompanies malignant progression. Accordingly, H3K27me3 loss has become diagnostically relevant in surgical pathology and is now routinely assessed by immunohistochemistry to support the distinction between atypical neurofibromas and MPNSTs [81,82]. Because PRC2 disruption leaves measurable mutational and epigenetic consequences, it also provides a strong biological rationale for translational efforts aimed at capturing methylation-based and chromatin-derived signatures beyond the tissue compartment [83,84].

As tumors progress further, additional disruption of canonical tumor suppressor pathways becomes common. Alterations affecting *TP53* and *RB1* are particularly relevant in advanced MPNSTs, where they further compromise genomic integrity and reinforce uncontrolled proliferation [39,85]. These changes are associated with higher proliferative indices, extensive chromosomal instability, and greater metastatic potential [86]. In a subset of tumors, amplification of oncogenic drivers such as *MET*, *PDGFRA*, or *EGFR* has also been reported, suggesting additional layers of biological heterogeneity and possible therapeutic vulnerability [87,88]. At this stage, the disease is typically characterized by greater genomic complexity and a larger tumor fraction, features that are also likely to influence the detectability of tumor-derived material in plasma [89].

This progressive accumulation of alterations unfolds within a highly heterogeneous tumor ecosystem. Recent single-cell and spatial transcriptomic studies have shown that MPNSTs are composed of multiple coexisting subclones with distinct molecular states [36,90]. Some regions may retain only earlier driver events, such as *NF1* and *CDKN2A* loss, whereas others acquire additional alterations in *SUZ12*, *EED*, and *TP53*, thereby gaining distinct proliferative capacities and therapeutic vulnerabilities [64,91]. This intratumoral heterogeneity has major implications for tissue diagnosis: localized biopsies may under-sample the most biologically relevant clone, miss focal areas of malignant progression, or overrepresent relatively quiescent regions that do not reflect the most aggressive disease component of the lesion [90,92].

In NF1 patients, who often harbor multiple anatomically distinct lesions at different stages of evolution, this sampling limitation becomes even more consequential.

Taken together, tissue-based studies define a coherent molecular sequence of progression from benign PN to MPNST, in which biallelic *NF1* loss establishes the permissive background, *CDKN2A/B* deletion marks early premalignant evolution, PRC2 inactivation drives epigenetic collapse and malignant transition [42], and *TP53*/*RB1* alterations accompany advanced genomic instability [93,94]. This tissue-defined roadmap is important not only for pathological classification, but also because it provides the biological framework for asking which transformation-associated alterations might later become detectable outside the tumor itself [42,68,75]. In this context, the molecular history reconstructed from tissue studies forms the essential bridge to the next section, in which circulating tumor DNA approaches will be examined as emerging tools to translate these discoveries into minimally invasive clinical surveillance strategies in NF1.

## 3. Liquid Biopsy Studies Across the NF1 Tumor Continuum

Building on the tissue-defined molecular roadmap outlined above, this section reviews the published liquid biopsy studies that have explored whether transformation-associated alterations can be detected in plasma across the NF1 tumor continuum, including benign PNs, ANNUBPs, and MPNSTs [36,41,42,91]. Based on the bibliography currently available, NF1-specific plasma evidence remains relatively limited and is focused mainly on cfDNA-based ultra-low-pass whole-genome sequencing (ULP-WGS) and fragmentomic analyses, rather than on broadly validated mutation-, methylation-, or RNA-based assays [42,53,89,94,95,96,97]. Accordingly, this section focuses on studies directly supported by the current reference list, while the biological determinants limiting ctDNA detection in NF1 are discussed separately in the next chapter.

Among the available NF1-specific studies, Szymanski et al. (2021) provided the strongest plasma-based evidence by applying ULP-WGS to cfDNA to distinguish MPNST from its benign precursor lesions [89]. Using copy-number profiling and inferred tumor fraction, the authors showed that plasma-derived genomic signals can discriminate malignant from benign disease states, and that in silico size selection can further enrich the tumor-derived signal and improve classification performance [89]. The chromosomal abnormalities identified in plasma were consistent with the known genomic landscape of MPNST, including recurrent gains involving chromosome arms 1q, 7p, 8q, 9q, and 17q, together with losses affecting tumor suppressor loci such as *CDKN2A/B* and *SUZ12* [70,89]. Moreover, plasma tumor fraction correlated with radiographic tumor burden, supporting the concept that cfDNA analysis may have value not only for cross-sectional classification but also for longitudinal disease monitoring [89].

Complementary evidence was provided by Mattox et al. (2022), who investigated plasma cfDNA in NF1-associated peripheral nerve sheath tumors using genome-wide aneuploidy scoring (GAS) together with focal copy-number analysis [98]. Their results showed that genome-wide aneuploidy analysis alone identified a subset of MPNSTs, whereas the inclusion of sub-chromosomal copy-number alterations improved sensitivity while maintaining high specificity. In a subset of patients, mutation analysis of plasma cfDNA also identified alterations in genes including *NF1*, *NF2*, *RB1*, *TP53BP2*, and *GOLGA2*, further supporting the feasibility of plasma-based detection of transformation-associated genomic events in NF1 [98].

A particularly relevant clinical question in NF1 is whether liquid biopsy can help resolve the intermediate biological state represented by ANNUBP, which is widely regarded as a premalignant lesion bridging benign PN and overt MPNST [41,63,68,91]. This issue is clinically relevant because NF1 patients often harbor multiple lesions at different stages of evolution, and malignant transformation may arise within spatially restricted nodular components rather than across the entire tumor mass [42,68]. In this setting, plasma-based assays are particularly attractive because they may capture transformation-associated genomic material shed by the biologically most active lesions without requiring repeated tissue sampling.

Whereas Szymanski et al. incorporated fragment-size enrichment within a ULP-WGS framework, Sundby et al. (2024) applied a dedicated plasma cfDNA fragmentomic approach to distinguish benign lesions, ANNUBPs, and MPNSTs in NF1 [53,89].

Their findings suggested that fragment size distributions and related fragmentomic features can improve discrimination across disease states, thereby complementing copy-number-based approaches and refining classification along the PN–ANNUBP–MPNST continuum [53]. More broadly, this observation is consistent with the wider oncology literature showing that genome-wide cfDNA fragmentation patterns encode tumor-associated biological information beyond sequence variation alone [84,99].

Epigenetic reprogramming also represents a relevant translational target for plasma-based assays in NF1. Tissue studies have shown that malignant progression is closely linked to PRC2 loss, depletion of H3K27me3, and methylation-based subgrouping of peripheral nerve sheath tumors [56,64,71,73,77,79,80,81,82]. These observations are now supported by plasma-based evidence. Tomczak et al. profiled plasma cfDNA methylation using cfMBD-seq (reagents by Roche and Diagenode; Illumina platform) and showed that patients with NF1-associated MPNST display a distinct hypermethylation pattern compared with individuals with NF1 without malignant transformation. In their study, 73 candidate MPNST-specific CpGs were identified, 63 of 67 selected genomic regions showed higher methylation in plasma from MPNST cases, and 15 CpG islands consistently distinguished confirmed MPNST from NF1 controls without malignant transformation, supporting the potential value of cfDNA methylation biomarkers for early detection in NF1 [100].

Circulating RNA biomarkers may also provide an additional layer of non-invasive molecular information in NF1. In a recent serum-based study, Napolitano et al. profiled circulating miRNAs in a cohort of 126 NF1 patients using small non-coding RNA sequencing followed by qRT-PCR (TaqMan assays, Applied Biosystems) validation and identified a six-miRNA signature that discriminated NF1 subjects from healthy controls. The validated signature included upregulation of miR-100-5p, miR-16-2-3p, miR-4508, and miR-885-5p, together with downregulation of miR-107 and miR-4433b-5p, and network analysis linked these miRNAs to ERK/MAPK, PI3K/AKT, and mTOR-related pathways. Although this study does not specifically address discrimination among PN, ANNUBP, and MPNST, it supports the broader concept that circulating RNA species may serve as informative biomarkers in NF1 and could complement future plasma-based surveillance strategies [101].

Overall, current evidence indicates that liquid biopsy in NF1 remains an emerging and methodologically heterogeneous field, in which ULP-WGS, aneuploidy-based approaches, and cfDNA fragmentomics currently provide the strongest disease-specific support for non-invasive detection of malignant transformation [48,82]. These strategies are particularly relevant because they are grounded in the known molecular progression from PN to ANNUBP to MPNST, including recurrent copy-number alterations and increasing genomic complexity [36,42,69,70,72,85,86,89,91]. By contrast, cfDNA methylation profiling and circulating RNA-based analytes represent promising complementary approaches, but their clinical role in NF1 surveillance still requires further validation in larger and biologically well-characterized cohorts [100,101].

Taken together, the available literature supports the idea that plasma-based assays may complement imaging and tissue evaluation in the longitudinal management of NF1-associated peripheral nerve sheath tumors, particularly for detecting malignant progression within a heterogeneous and multifocal disease spectrum [42,47,48,49,52,89]. However, the clinical performance of these approaches is strongly influenced by biological factors, including limited tumor DNA release, lesion-specific shedding dynamics, low tumor fraction, and marked intrapatient heterogeneity, which are examined in the following section on biological barriers to detection [90,94,96,97,102,103,104].

## 4. Biological Barriers to ctDNA Detection in NF1

The clinical promise of liquid biopsy in NF1 is fundamentally shaped by the biology of the disease itself. In NF1, the main obstacle is not merely the analytical sensitivity of the assay, but the fact that tumor-derived DNA often enters the circulation in very small, highly variable, and biologically inconsistent amounts. This limitation is especially relevant because NF1-associated peripheral nerve sheath tumors do not represent a single static category, but rather evolve along a heterogeneous pathological continuum that includes PNs, ANNUBP, and MPNSTs [41,42,63,69,72]. Within this continuum, ctDNA release is not uniform across lesions or across stages of progression. As a result, the absence of a detectable plasma signal cannot be interpreted straightforwardly as absence of biological activity, because detectability depends first on whether a given lesion is shedding enough tumor-derived material to become systemically visible.

A central biological barrier is therefore the extremely low fraction of ctDNA relative to total cfDNA. Across solid tumors, ctDNA often represents only a minute fraction of plasma cfDNA, particularly in localized disease and in tumors with limited shedding capacity [96,99,105,106,107,108]. In NF1, this problem is magnified by the biology of PNs, which frequently account for much of the disease burden and yet often remain relatively indolent, slow-growing, and biologically quiet for prolonged periods. Their architecture is characterized by abundant extracellular matrix, a substantial stromal compartment, and a complex non-neoplastic microenvironment enriched in mast cells and other supporting elements, all of which shape tumor behavior and may further hinder the effective release and diffusion of tumor DNA into the bloodstream [9,58,60,62,109,110]. In practical terms, this means that even patients with a large cumulative tumor burden may still have very little detectable ctDNA when disease is driven primarily by stable benign lesions.

This low-shedding state is closely linked to the biological mechanisms by which ctDNA is generated. Circulating tumor DNA does not arise through a single passive pathway, but instead reflects a dynamic interplay of apoptosis, necrosis, and other DNA release-associated processes, followed by fragmentation and degradation in the circulation [102,103,108,111]. Against this background, benign PNs are intrinsically disadvantaged as ctDNA sources because they generally show limited proliferative activity and relatively little tissue breakdown, thereby contributing only minimally to the plasma tumor DNA pool [38,39,42]. By contrast, MPNSTs are biologically more compatible with measurable ctDNA release because they are associated with more aggressive growth, greater genomic instability, and more frequent necrotic change [39,44,85]. Yet even this transition does not guarantee reliable plasma detectability. Deeply located tumors, lesions with limited vascular access, or masses arising in anatomically constrained sites such as paraspinal or retroperitoneal compartments may still release DNA inefficiently into the systemic circulation [112,113]. The biological consequence is a recurrent paradox in NF1: large lesions may remain molecularly silent in plasma, whereas smaller but more aggressive lesions may become detectable only once transformation is already more advanced.

This helps explain why ctDNA burden in NF1 does not necessarily track with tumor volume in a simple linear manner. Plasma detectability is influenced less by size alone than by a combination of biological features, including proliferative rate, cell death dynamics, stromal density, vascularization, and anatomical location. The additional influence of physiological clearance further accentuates this disconnect. Circulating DNA fragments have a short half-life and are rapidly removed through nuclease activity and systemic clearance pathways, meaning that measured ctDNA levels reflect a transient equilibrium between release and elimination rather than a stable proxy for tumor mass [102,108,114,115]. In slowly evolving NF1-associated lesions, this equilibrium may remain persistently shifted toward undetectable circulating fractions. Accordingly, a low or absent plasma signal may simply indicate that the biological conditions required for efficient shedding have not been met, rather than that the lesion is clinically irrelevant.

The problem becomes even more pronounced in intermediate lesions along the PN-to-ANNUBP-to-MPNST trajectory. These lesions may already harbor progression-associated molecular changes, including *CDKN2A/B* loss and increasing genomic complexity, while still lacking the degree of proliferative acceleration, necrosis, or vascular remodeling typically associated with robust ctDNA release [63,68,69,72,91]. In this setting, early clonal evolution may remain spatially confined to restricted subregions of a lesion and therefore fail to generate a systemic signal strong enough to cross detection thresholds. This is a particularly important point in NF1 surveillance, because the biologically most consequential phase of transformation may also be the one in which plasma-based detection is least dependable. The same lesion may already be evolving at the molecular level yet remain effectively invisible in blood because the emerging clone is too localized, too small, or too poorly shed to be represented in plasma.

Emerging studies in NF1 liquid biopsy are consistent with this biological model. Plasma-based approaches appear to perform better in more advanced or biologically active disease, whereas detection in premalignant or indolent lesions is less consistent and remains strongly influenced by tumor fraction and shedding dynamics [53,89,98,100]. Taken together, these findings suggest that the major biological limitation of ctDNA analysis in NF1 is not simply scarcity, but biological unevenness: some lesions shed very little because they are histologically benign and microenvironmentally constrained, whereas others may shed more only after clinically significant transformation has already progressed.

Taken together, these considerations define the core biological challenge of ctDNA detection in NF1. Tumor-derived DNA release is conditioned by lesion type, microenvironmental composition, anatomical context, and stage of progression, and for this reason plasma negativity should be interpreted with caution rather than reassurance. In NF1, a negative ctDNA result may reflect true biological quiescence, but it may equally reflect insufficient shedding from a stromal-rich lesion, a deep-seated tumor, or an evolving subclone that remains spatially restricted. Keeping this biological background is essential when considering the subsequent analytical and clinical aspects of liquid biopsy, because it helps explain why even highly sensitive downstream approaches may still struggle to capture the earliest and most clinically actionable phases of malignant transformation.

## 5. Analytical Challenges and Interpretative Pitfalls

Building on the biological barriers discussed in the previous section, the clinical application of liquid biopsy in NF1 is further complicated by technical and analytical factors that can substantially influence signal interpretation. In this context, the challenge is not limited to detecting circulating tumor-derived material, but also involves understanding whether the selected assay is capable of capturing the molecular features that are most informative for malignant progression. This point is particularly relevant in NF1-associated peripheral nerve sheath tumors, where progression may be more faithfully reflected by increasing copy-number complexity and epigenetic deregulation than by isolated plasma variants alone [64,92,116].

A major analytical consequence of this setting is the risk of false-negative results. In practice, a negative plasma finding cannot be interpreted as evidence against biologically relevant progression, since nondetection may reflect very low tumor fraction, limited molecular input, stochastic sampling effects, insufficient library complexity, or panel-design constraints rather than a true absence of tumor-derived material. This is especially important in NF1, where low-shedding lesions may generate circulating signals close to the assay-specific limit of detection [89].

At the same time, low-signal settings are also vulnerable to false-positive interpretation. At very low allele fractions, background errors introduced during amplification, library preparation, or sequencing can be difficult to distinguish from genuine tumor-derived events. In addition, plasma-only analyses are intrinsically exposed to biological confounders, most notably clonal hematopoiesis, which may generate apparently somatic cfDNA variants that do not originate from the tumor. As shown by Razavi et al. [104], matched leukocyte sequencing therefore represents one of the most robust strategies for distinguishing true tumor-derived alterations from germline and clonal hematopoiesis-related findings, particularly when plasma variants are detected near the analytical threshold [117].

These limitations explain the interest in highly sensitive, error-suppressed sequencing strategies. Approaches such as cancer personalized profiling by deep sequencing (CAPP-Seq) and its iDES-enhanced form, described by Newman et al. [118,119] (using Roche NimbleGen capture and IDT molecular identifiers), improve rare-event detection by suppressing background noise and increasing analytical sensitivity. However, analytical sensitivity alone does not guarantee biological interpretability, especially when variants are detected close to assay-specific calling thresholds [117] (e.g., Twist Bioscience custom panels). Even high-depth, error-suppressed sequencing does not fully resolve the more fundamental issue that, in NF1, the most informative circulating readouts may not always be mutation-based [119].

For this reason, mutation-independent approaches may be particularly valuable in NF1. Fragmentomic readouts are relevant because they are less dependent on the detection of individual low-frequency variants and may better capture genome-wide correlates of malignant progression. In this respect, Szymanski et al. [89] and Sundby et al. [53] support the analytical value of cfDNA fragmentation-based signals in distinguishing benign, premalignant, and malignant peripheral nerve sheath tumors. A similar rationale applies to methylation-based approaches, which may provide a biologically coherent alternative when variant-based plasma detection is constrained by low tumor fraction or marked heterogeneity, as supported by the plasma methylation data reported by Tomczak et al. [100]. By contrast, circulating microRNAs are better regarded as exploratory complementary biomarkers than as direct surrogates of cfDNA-based tumor monitoring [120].

Overall, the analytical challenge in NF1 cannot be reduced to sequencing depth alone. Rather, it reflects the combined effect of weak circulating signals, rare-event noise, biological confounders, and the limited ability of mutation-only assays to fully represent the molecular architecture of progression. For this reason, the most promising analytical strategy is likely to rely on the integration of complementary signal layers rather than on exclusive dependence on mutation-based ctDNA calling. At the same time, the performance and reproducibility of any of these approaches remain critically dependent on pre-analytical and workflow variables, which are addressed in the following section [121].

## 6. Pre-Analytical Variables and Workflow Standardization

The clinical value of the analytical strategies discussed above depends heavily on the control of pre-analytical variables, which remain one of the main sources of variability in cfDNA testing [121,122,123]. This aspect becomes especially important in NF1-associated peripheral nerve sheath tumors, where the circulating tumor-derived signal may already be weak and can therefore be affected quite substantially by suboptimal sample handling [89]. It is also important to remember that plasma cfDNA is not composed exclusively of tumor-derived fragments. In most cases, ctDNA represents only a fraction of total cfDNA, and any additional release of high-molecular-weight genomic DNA from lysed white blood cells may dilute the tumor-derived component, reducing the effective tumor fraction available for downstream analysis [122,123]. From a practical perspective, this is one of the main reasons why plasma is generally preferred over serum, and why the choice of blood collection tube should be matched to the expected time to processing. When samples can be processed rapidly, standard EDTA tubes are usually acceptable. By contrast, when delays in transport or laboratory handling are expected, cell-stabilizing tubes may help preserve cfDNA integrity by limiting leukocyte lysis during the pre-analytical interval [121,122,124].

Plasma processing also requires careful attention. Sequential centrifugation steps are commonly used to reduce residual cellular material and minimize genomic DNA contamination, thereby improving the purity of the cfDNA input for downstream assays [122,123]. In a low-input setting such as NF1, the amount of plasma available for extraction may itself influence the probability of recovering sufficient genome equivalents for rare-event detection, making blood volume and plasma yield relevant considerations already at the earliest stages of the workflow [123,125]. Pre-analytical rigor, however, does not end with centrifugation. Aliquoting strategy, storage conditions, and repeated freeze–thaw cycles may all affect cfDNA integrity and reproducibility, particularly when the tumor-derived component is already close to the assay-specific limit of detection [122,125]. In this context, sample processing should not be regarded as a merely technical step before sequencing, but rather as one of the factors that directly shape the biological reliability of the final result.

Extraction and input quality assessment are equally important. Because cfDNA is typically enriched in short nucleosomal fragments, extraction methods and library preparation workflows should be selected with the awareness that both yield and fragment-size bias can vary substantially across protocols. In practical terms, this means that two workflows starting from the same plasma volume may still produce very different downstream performance if they differ in extraction efficiency, fragment recovery, or compatibility with low-input sequencing. Basic quality control steps therefore remain highly informative, especially for readers approaching this field for the first time. Fluorometric quantification is generally preferred over spectrophotometric methods for low-concentration DNA, while fragment-size assessment by capillary electrophoresis can help determine whether the extracted material is consistent with the expected cfDNA profile or instead suggests contamination by high-molecular-weight genomic DNA [122,125]. Even when these steps are not described in detail, they have a major impact on the reproducibility and interpretability of liquid biopsy data.

For clinical application, this means that, in NF1, pre-analytical rigor should not be regarded as a purely procedural issue, but rather as a necessary condition for obtaining biologically meaningful liquid biopsy results [121,123]. In a disease setting where the circulating signal may already be scarce, inadequate handling at the wet-bench level can easily undermine even the most sophisticated downstream analytical platforms [89,122]. Standardization of blood collection, plasma processing, storage, extraction, and input quality control should therefore be considered a core component of any NF1 liquid biopsy workflow, and not simply a technical prelude to sequencing. For its incorporation into routine surveillance, this level of pre-analytical standardization becomes even more relevant, as liquid biopsy should not be interpreted as a stand-alone test.

## 7. Future Perspectives and an Integrated Surveillance Model

At present, liquid biopsy alone is unlikely to replace the tools currently used for NF1 surveillance. A more realistic view is that it may become one component of a broader and more biologically informative surveillance model. The studies available so far show that plasma-based assays can capture transformation-associated signals in at least a subset of patients, but they also show that these signals are often weak, heterogeneous, and strongly influenced by tumor fraction, lesion-specific shedding, and the multifocal nature of the disease [53,89,100]. Thus, the question is no longer only whether ctDNA-related information can be detected in NF1, but how that information can be interpreted and used in a clinically meaningful way. This is particularly relevant in NF1, where surveillance is often far from straightforward. Patients may harbor multiple lesions with different biological behavior, and the lesions that appear most suspicious on imaging are not always the easiest ones to sample or follow over time [47]. Imaging remains essential, but it does not always resolve biological uncertainty. Tissue biopsy also remains indispensable, but it may be affected by sampling bias, especially in large or heterogeneous tumors. In this setting, the value of liquid biopsy may be less in replacing imaging or histopathology, and more in adding a molecular layer that can be assessed over time. Clinically, this implies that liquid biopsy could help support earlier recognition of malignant progression, identify patients or lesions requiring closer radiological attention, guide prioritization for tissue sampling, and provide complementary information when repeated invasive procedures are not feasible [53,89].

From a surveillance perspective, future strategies in NF1 will probably depend on multimodal approaches in which imaging, tissue biopsy, and liquid biopsy are interpreted together rather than separately. Imaging will remain central for anatomical definition, lesion burden assessment, and longitudinal follow-up. Tissue biopsy will continue to provide the histopathological resolution required for definitive classification. Liquid biopsy, in contrast, may contribute dynamic information on tumor-associated genomic and epigenetic changes, particularly if approaches based on copy-number profiling, fragmentomics, and methylation can be further standardized and validated in larger prospective cohorts [47,53,100]. In this context, the goal may be less to identify a single ideal biomarker and more to understand how different readouts can be combined to improve risk stratification along the PN–ANNUBP–MPNST continuum. What is still missing, however, is not only larger case series but also better biological annotation of lesions, harmonized pre-analytical and analytical workflows, and longitudinal studies designed around clinically relevant endpoints. This will be essential to understand not only whether a circulating signal can be detected, but also when it becomes informative, how it correlates with imaging and pathology, and whether it can truly improve clinical decision-making [47,121]. If these conditions are met, liquid biopsy could become a useful component of integrated surveillance in NF1, not because it replaces current standard tools, but because it may help connect morphological change with the underlying biological progression of disease.

## Data Availability

No new data were created or analyzed in this study.

## Abbreviations

The following abbreviations are used in this manuscript:

NF1	Neurofibromatosis type 1
MPNST	Malignant peripheral nerve sheath tumor
PN	Plexiform neurofibroma
PET/CT	Positron emission tomography/computed tomography
18F-FDG	18F-Fluorodeoxyglucose
ctDNA	Circulating tumor DNA
cfDNA	Cell-free DNA
GISTs	Gastrointestinal stromal tumors
ANNUBP	Atypical neurofibromatous neoplasm of uncertain biologic potential
PRC2	Polycomb repressive complex 2
MRI	Magnetic resonance imaging
SUV	Standardized uptake value
SCF	Stem cell factor
PDGF	Platelet-derived growth factor
PDGFRA	Platelet-derived growth factor alpha
PNSTs	Peripheral nerve sheath tumors
ULP WGS	Ultra-low-pass whole-genome sequencing
GAS	Genome-wide aneuploidy scoring
CAPP-Seq	Cancer personalized profiling by deep sequencing
